# Hfq-Assisted RsmA Regulation Is Central to *Pseudomonas aeruginosa* Biofilm Polysaccharide PEL Expression

**DOI:** 10.3389/fmicb.2020.482585

**Published:** 2020-11-17

**Authors:** Yasuhiko Irie, Agnese La Mensa, Victoriia Murina, Vasili Hauryliuk, Tanel Tenson, Victoria Shingler

**Affiliations:** ^1^Institute of Technology, University of Tartu, Tartu, Estonia; ^2^Department of Molecular Biology, Umeå University, Umeå, Sweden; ^3^The Laboratory for Molecular Infection Medicine Sweden (MIMS), Umeå University, Umeå, Sweden

**Keywords:** *Pseudomonas aeruginosa*, RsmA, Vfr, FleQ, Hfq

## Abstract

To appropriately switch between sessile and motile lifestyles, bacteria control expression of biofilm-associated genes through multiple regulatory elements. In *Pseudomonas aeruginosa*, the post-transcriptional regulator RsmA has been implicated in the control of various genes including those related to biofilms, but much of the evidence for these links is limited to transcriptomic and phenotypic studies. RsmA binds to target mRNAs to modulate translation by affecting ribosomal access and/or mRNA stability. Here, we trace a global regulatory role of RsmA to inhibition of the expression of Vfr—a transcription factor that inhibits transcriptional regulator FleQ. FleQ directly controls biofilm-associated genes that encode the PEL polysaccharide biosynthesis machinery. Furthermore, we show that RsmA alone cannot bind *vfr* mRNA but requires the assistance of RNA chaperone protein Hfq. This is the first example where a RsmA protein family member requires another protein for binding to its target RNA.

## Introduction

The opportunistic pathogen *Pseudomonas aeruginosa* can be isolated from a wide range of environmental niches (Crone et al., 2020), in large part owing to its versatile metabolic capabilities (Arai, 2011). It is proficient in colonizing various eukaryotic organisms (Rahme et al., 1995) and can cause both acute and chronic infections, with the latter often associated with biofilm-like modes of growth at the sites of infection (Gellatly and Hancock, 2013). *P. aeruginosa* is highly competitive against other microbial species, with its adaptability and competitive fitness being aided by the production of various secondary metabolites and virulence factors (Tashiro et al., 2013; Khare and Tavazoie, 2015). Many of these cellular processes are inter-regulated by multiple regulatory pathways, presumably to provide appropriate response mechanisms to a wide range of environmental cues. One of the central regulators that determine *P. aeruginosa* behavior is the post-transcription factor RsmA (Regulator of Secondary Metabolites) as evidenced by its null mutant phenotypes linked to motility (Heurlier et al., 2004), virulence (Mulcahy et al., 2006, 2008; O’Grady et al., 2006), biofilm (Mulcahy et al., 2006; Irie et al., 2010), growth (Irie et al., 2010), and secreted products (Pessi et al., 2001; Heurlier et al., 2004).

RsmA belongs to the RsmA/CsrA family of dimeric RNA-binding proteins (Gutiérrez et al., 2005; Rife et al., 2005; Schubert et al., 2007). RsmA/CsrA homologs are found across a wide range of both Gram-negative and Gram-positive bacterial species: in some species including *Escherichia coli*, they are called CsrA, while in other species including *P. aeruginosa*, they are named RsmA (White et al., 1996). RsmA/CsrA proteins inhibit translation of target mRNAs by binding to the ribosome binding sites, typically overlapping either the Shine–Dalgarno sequence or the start codon (Romeo et al., 2013). However, in a few cases identified thus far, they can also serve as positive regulators of translation by altering the secondary structures of bound RNAs (Patterson-Fortin et al., 2013; Ren et al., 2014) or protecting RNAs from degradation (Yakhnin et al., 2013).

Many RsmA/CsrA-targeted mRNAs are reported to have altered RNA decay rates. Several studies attempted to exploit this property to determine the global *P. aeruginosa* RsmA regulon using microarrays (Lawhon et al., 2003; Burrowes et al., 2006; Brencic and Lory, 2009). However, this approach cannot distinguish directly regulated genes from indirectly regulated gene sets that result from cascade regulation, nor can it identify target RNAs that do not exhibit changes in their stability (Baker et al., 2007; Pannuri et al., 2012). In *P. aeruginosa*, direct RsmA-mediated translation inhibition has been demonstrated for the *P. aeruginosa*-specific biofilm polysaccharide operon *psl* without any effect on the level of transcription (Irie et al., 2010). Hence, the *psl* operon was not prominently featured in transcriptomic studies despite a strong biofilm phenotype (Burrowes et al., 2006; Brencic and Lory, 2009), raising concerns of what is currently considered to be “the RsmA regulon” of *P. aeruginosa*. To address this concern, more direct global analyses of RsmA/CsrA using techniques such as ChIP-Seq, CLIP-Seq, RIP-Seq, and ribosome profiling have recently been performed (Holmqvist et al., 2016; Potts et al., 2017; Sahr et al., 2017; Gebhardt et al., 2020).

Here, we present genetic and biochemical evidence that RsmA serves a master regulatory role through several intermediate transcription factors that are known to control different cellular processes. Through cascade regulation, originating from a direct effect on *vfr* mRNA, RsmA indirectly affects biofilm polysaccharide expression. Furthermore, we discovered an unexpected regulatory interplay between RsmA and the RNA chaperone protein Hfq on the target mRNA, whereby RsmA could only bind in the presence of Hfq. To our knowledge, this is the first example of RsmA/CsrA being unable to bind to its RNA target unless assisted by another protein.

## Results

### RsmA Is a Post-transcriptional Regulator of *vfr* Through Which *fleQ* and *pel* Transcripts Are Indirectly Regulated

*Pseudomonas aeruginosa* produces at least three different extracellular biofilm polysaccharides: alginate, PEL, and PSL (Ryder et al., 2007). PEL and PSL are co-regulated by several factors including the secondary messenger molecule c-di-GMP via the c-di-GMP-binding transcriptional regulator FleQ (Hickman and Harwood, 2008) and possibly also quorum sensing (Sakuragi and Kolter, 2007; Gilbert et al., 2009). Similar to PSL, PEL has long been considered to be regulated by RsmA (Gooderham and Hancock, 2009) based on a model originally proposed from a microarray study (Goodman et al., 2004). However, unlike the *psl* operon transcript, which was genetically and biochemically demonstrated to be directly regulated by RsmA (Irie et al., 2010), no direct evidence for the *pel* operon has been presented.

**FIGURE 1:**
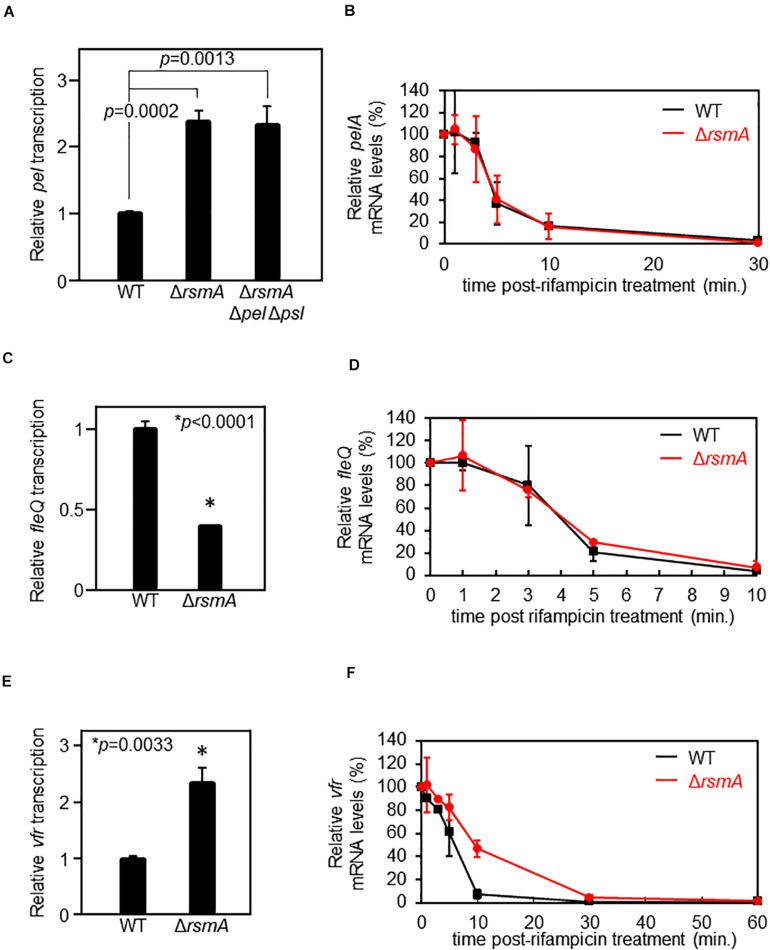
RsmA does not directly regulate *pel* nor *fleQ* but represses *vfr* by altering mRNA stability. **(A)** Single-copy transcriptional *lacZ* fusion constructs reveal up-regulation of *pel* transcripts in the Δ*rsmA* background as compared with wild type (WT). The Δ*rsmA* strain overexpresses the PSL polysaccharides, which causes an elevation of intercellular c-di-GMP (Irie et al., 2012). Thus, we also tested a Δ*rsmA* Δ*pel* Δ*psl* strain, which has low c-di-GMP due to the absence of PSL (Irie et al., 2012). Note that *pel* is equally up-regulated in the Δ*rsmA* Δ*pel* Δ*psl* strain, demonstrating that the *pel* transcriptional phenotype of Δ*rsmA* is PEL-, PSL-, and c-di-GMP-independent. The *y*-axis values are expressed as relative to WT as 1. **(B)** Real-time quantitative PCR (qPCR) at different time points after rifampicin treatment shows indistinguishable stabilities of the *pelA* transcript in the WT and Δ*rsmA* strains. The *y*-axis values are expressed as relative to *t* = 0 as 100%. **(C)** Single-copy transcriptional *lacZ* fusion constructs reveal down-regulation of *fleQ* transcripts in the Δ*rsmA* background as compared with WT. The *y*-axis values are expressed as relative to WT as 1. **(D)** qPCR at different time points after rifampicin treatment shows indistinguishable stabilities of the *fleQ* transcript in the WT and Δ*rsmA* strains. The *y*-axis values are expressed as relative to *t* = 0 as 100%. **(E)** Single-copy transcriptional *lacZ* fusion constructs reveal up-regulation of *vfr* transcripts in the Δ*rsmA* background as compared with WT. The *y*-axis values are expressed as relative to WT as 1. **(F)** qPCR at different time points after rifampicin treatment show that *vfr* mRNA is less stable in WT as compared with the Δ*rsmA* strain. The *y*-axis values are expressed as relative to *t* = 0 as 100%.

Consistent with a previous microarray study (Brencic and Lory, 2009), using a chromosomal transcriptional reporter, we found that *pel* is up-regulated in the Δ*rsmA* mutant as compared with the wild-type (WT) background (Figure 1A). However, the PSL polysaccharides are also over-produced in a Δ*rsmA* strain (Irie et al., 2010), resulting in elevated intracellular levels of c-di-GMP due to PSL signaling (Irie et al., 2012). Given that the *pel* genes are transcriptionally up-regulated by c-di-GMP (Hickman and Harwood, 2008; Baraquet et al., 2012) and the Δ*rsmA* strain has elevated c-di-GMP levels (Irie et al., 2012), it is plausible that the up-regulation of *pel* could be a consequence of increased c-di-GMP levels. To uncouple *pel* expression from elevated c-di-GMP, we also measured *pel* transcriptional reporter activities when introduced into a low c-di-GMP Δ*rsmA* Δ*pel* Δ*psl* triple mutant background (Irie et al., 2012). In this strain, *pel* is still up-regulated (Figure 1A), indicating that the up-regulation is caused by lack of RsmA rather than changes in intracellular c-di-GMP concentration imposed by RsmA regulation of diguanylate cyclases and/or phosphodiesterases.

Since direct regulation of mRNA levels by RsmA/CsrA often acts via altered mRNA stability (Romeo et al., 2013), we tested whether the apparent *pel* transcriptional changes between the WT and Δ*rsmA* strains is due to altered *pel* mRNA stability. This, however, is not the case (Figure 1B), suggesting that the differences in *pel* expression are likely due to altered activity of the promoter. Because the RNA-binding protein RsmA is not a transcription factor, we tested the possibility of RsmA affecting expression of a known transcriptional regulator of *pel*, namely, FleQ. As a well-characterized transcriptional activator of *pel* (Hickman and Harwood, 2008; Baraquet et al., 2012), potential RsmA-mediated alterations in expression of FleQ would be anticipated to affect transcription of the *pel* genes. We found that a transcriptional reporter fusion of *fleQ* gave lower levels in the absence of RsmA (Figure 1C). This is consistent with previous data showing *pel* transcript levels are up-regulated in Δ*fleQ* backgrounds (Hickman and Harwood, 2008).

The data above could lend themselves to the interpretation that RsmA serves as a positive regulator of *fleQ*. While RsmA is more commonly known to be a translational repressor, this possibility is not without precedence. Examples of RsmA/CsrA proteins stimulating translation of target mRNAs include *phz2* in *P. aeruginosa* (Ren et al., 2014) and *moaA* and *flhDC* in *Escherichia coli* (Patterson-Fortin et al., 2013; Yakhnin et al., 2013). While *P. aeruginosa* FleQ and *E. coli* FlhD_4_C_2_ share no sequence or mechanistic similarities, they are functional counterparts, with both being class I master regulators of flagellar biosynthesis in their respective organisms (Dasgupta et al., 2003). In addition, flagellar motility was previously found to be positively controlled by RsmA in *P. aeruginosa* (Heurlier et al., 2004), while in *E. coli*, CsrA has been shown to up-regulate flagellar gene expression through protection of *flhDC* mRNA from degradation (Yakhnin et al., 2013). It follows that if RsmA regulated *fleQ* in an analogous manner, *fleQ* mRNAs would be stabilized by RsmA and, thus, less stable in the null mutant. However, no evidence of altered mRNA turnover rate was found between WT and Δ*rsmA* strains (Figure 1D). Therefore, we hypothesized that the direct action of RsmA may lie even further upstream in a regulatory cascade that involved control of *fleQ* by Vfr.

Vfr acts as a transcriptional repressor of the *fleQ* promoter (Dasgupta et al., 2002). Therefore, to be consistent with the above phenotypes, RsmA would have to serve as a repressor of *vfr*. In line with this idea, comparison of *vfr* transcriptional reporter activities in WT and Δ*rsmA* strains revealed up-regulation of *vfr* transcripts in the absence of RsmA (Figure 1E and Supplementary Figure S1). In contrast to *pel* (Figure 1B) and *fleQ* (Figure 1D), the transcript stability of *vfr* is significantly different between WT and Δ*rsmA* strains, such that *vfr* mRNAs are more stable in the absence of RsmA (Figure 1F). These results indicate that RsmA affects *vfr* mRNA levels by mediating changes in turnover rates.

### RsmA-Mediated Regulation of Vfr Alters Swimming and Twitching Motility

*Pseudomonas aeruginosa* uses at least two major modes of motility: flagellar-driven swimming motility through liquid and surface “twitching” motility using Type IV pilus (Burrows, 2012; Kazmierczak et al., 2015). Bacterial migration is an important aspect during *P. aeruginosa* biofilm development, as initial attachment to a surface is thought to require flagella, and mature microcolony formation has been attributed to Type IV pilus functions (O’Toole and Kolter, 1998; Klausen et al., 2003). Thus, fine-tuned regulatory control of motility and biofilm genes is a necessity for effective *P. aeruginosa* adaptation, particularly when switching between motile and sessile lifestyles.

**FIGURE 2:**
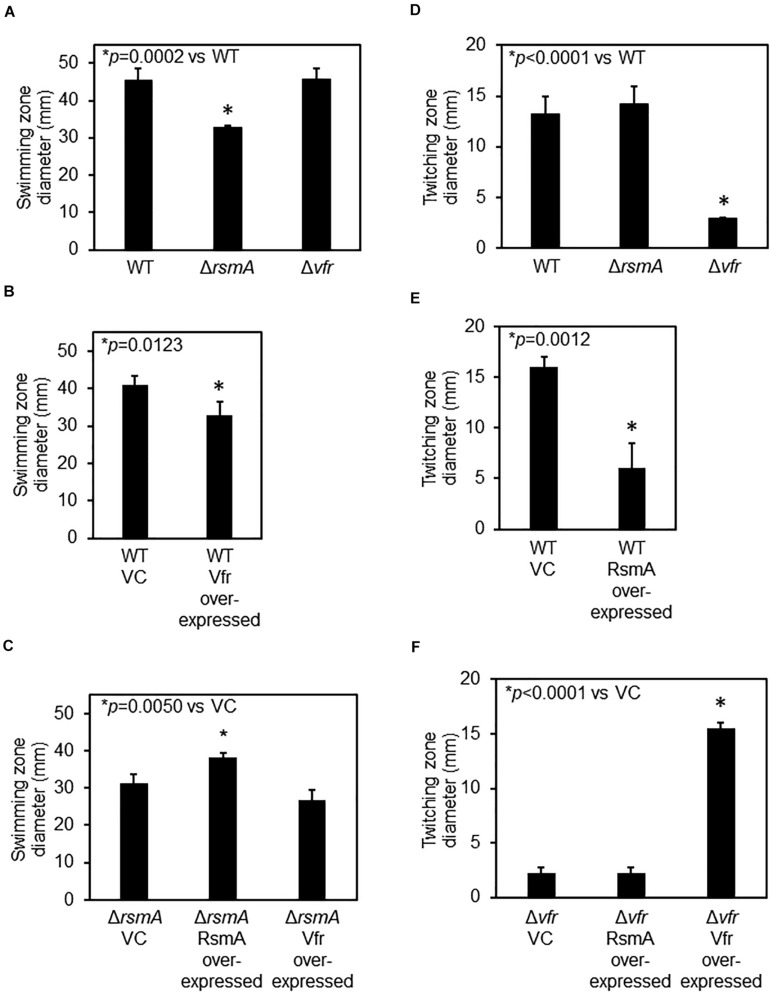
RsmA regulatory cascade affects flagellar and Type IV pilus-mediated motilities. **(A)** Flagellar-mediated swimming motility is decreased for the Δ*rsmA* strain but not the Δ*vfr* strain. **(B)** Vfr over-expression leads to decreased swimming motility. VC, vector control. Motility assays for VC and Vfr over-expressing strains were performed in the presence of 300 μg⋅ ml^–1^ of carbenicillin to ensure maintenance of the plasmids. **(C)** There is no statistical significance difference between the swimming motility of Δ*rsmA* and Vfr over-expression strains, indicating that Vfr is epistatically downstream of and in the same regulatory pathway as RsmA. Motility assays for these plasmid-containing strains were performed in the presence of 300 μg⋅ ml^–1^ of carbenicillin. **(D)** Type IV pilus-mediated twitching motility is reduced for Δ*vfr* as compared with wild type (WT). **(E)** RsmA over-expression leads to decreased twitching motility. Motility assays for VC and the RsmA over-expressing strain were performed in the presence of 300 μg⋅ ml^–1^ of carbenicillin. **(F)** RsmA over-expression in Δ*vfr* does not affect the low twitching motility of the parental Δ*vfr*. Motility assays for these plasmid-containing strains were performed in the presence of 300 μg⋅ ml^–1^ of carbenicillin.

Because FleQ is a class I master regulator of flagellar genes (Dasgupta et al., 2003) and RsmA and Vfr lie upstream of FleQ in the regulatory cascade, lack of either protein should predictively impact flagellar motility, but in opposite ways. As would be predicted, Δ*rsmA* and Vfr over-expressing strains have reduced flagellar (swimming) motilities (Figures 2A,B). Swimming motility level of Δ*vfr* is comparable with that of the WT (Figure 2A) probably because de-repression of flagellar genes does not necessarily lead to functional hyper-motility. There was no additive effect of over-expressing Vfr in the Δ*rsmA* background (Figure 2C), consistent with our model of a direct regulatory cascade from RsmA ⊣ Vfr ⊣ FleQ. Although expression of both the PEL (Figure 1A) and PSL (Irie et al., 2010) polysaccharides are up-regulated in Δ*rsmA*, expression of these two polysaccharides did not affect flagellar motility significantly (Supplementary Figure S2).

Mutants of *vfr* have previously been documented to be defective in Type IV pilus-dependent twitching motility (Whitchurch et al., 1996; Beatson et al., 2002). This is due to the positive regulation of the AlgZR two-component system by Vfr (Luo et al., 2015; Pritchett et al., 2015), which is required for the expression of Type IV pilus genes (Lizewski et al., 2004; Belete et al., 2008). As shown in Figures 2D,E, both the Δ*vfr* and RsmA over-expressing strains exhibited defects in twitching motility. Twitching motility of the Δ*rsmA* strain was comparable with that of WT. The latter is probably because, like de-repression of flagellar genes does not necessarily lead to functional hyper-swimming motility, de-repression of pilin genes also does not necessarily lead to functional hyper-twitching motility. Over-expressing RsmA in the Δ*vfr* background had no effect (Figure 2F) due to RsmA lying upstream of Vfr.

### Loss of RsmA Results in an Increased Acute Virulence Phenotype

**FIGURE 3:**
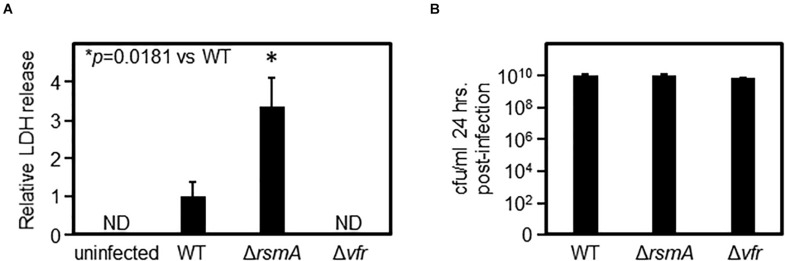
Δ*rsmA* displays an increased virulence phenotype against tissue culture cells. **(A)** After retinal pigment epithelial (RPE) cells were infected by *Pseudomonas aeruginosa* strains for 24 h, the levels of lactate dehydrogenase (LDH) in the media were measured. The increase of LDH released into the media by Δ*rsmA*-infected cells as compared with WT-infected cells indicates increased RPE cell lysis and, therefore, increased cytotoxicity caused by the Δ*rsmA* strain. Δ*vfr* is defective in this virulence assay, as no LDH could be detected in the media. The *y*-axis values are expressed as relative to WT as 1. ND, not detected. **(B)** Bacteria were retrieved from the wells of infected RPE cells, plated on agar media from a serial dilution series, and subsequently enumerated by counting colonies to determine colony-forming units (cfu) per milliliter. The identical cfu⋅ ml^–1^ for all three strains demonstrate that the differences in virulence as measured by LDH release in panel **(A)** is not due to an imbalance in bacterial loads.

Vfr is a cAMP-binding transcription factor that positively regulates acute virulence factors, including the Type III secretion system (Wolfgang et al., 2003). Vfr activates expression of the transcription factor ExsA, which in turn is required for the expression of the Type III secretion machinery (Marsden et al., 2016). In light of the data in Figures 1, 2, suggesting that RsmA is a direct negative regulator of *vfr* expression, it became of interest to test if the Δ*rsmA* mutant had enhanced virulence against eukaryotic cells due to increased expression of Vfr and therefore activation of virulence factors. To test this idea, we monitored cytotoxicity against eukaryotic cells by measuring lactate dehydrogenase (LDH) activity in the supernatants. LDH is a eukaryotic cytoplasmic protein and is released into the supernatant upon cell death/lysis. After 24-hour infection of human retinal pigment epithelial (RPE) cells, we observed increased cytotoxicity toward the RPE cells that were infected with Δ*rsmA* as compared with WT (Figure 3A). On the other hand, Δ*vfr* was attenuated in this respect as would be expected (Wolfgang et al., 2003). This pathogenicity phenotype was independent of bacterial load since equal numbers of bacteria were recovered from the same supernatant for WT, Δ*rsmA*, and Δ*vfr* (Figure 3B). The Δ*rsmA* cytotoxicity phenotype is consistent with a previous report (Mulcahy et al., 2006) and further supports the idea that effects of RsmA through Vfr are propagated to downstream processes in regulatory cascades.

### Hfq Is Required for RsmA to Bind to *vfr* mRNA

**FIGURE 4:**
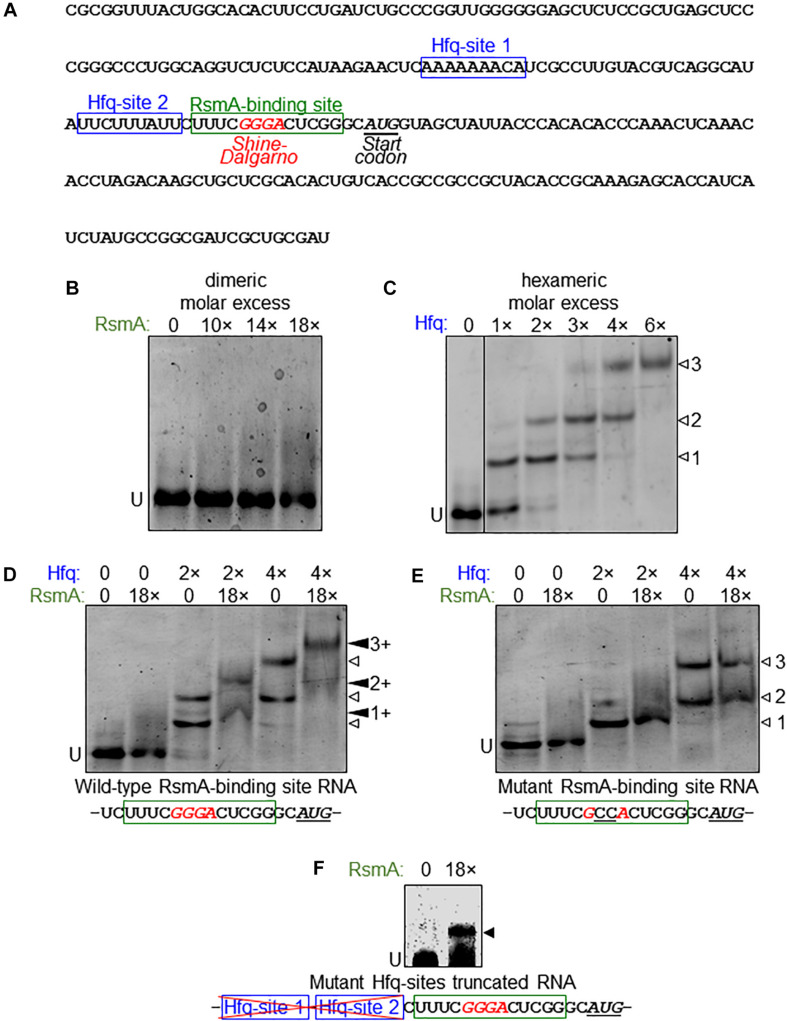
Direct binding of *vfr* mRNA by RsmA requires Hfq. **(A)** Sequence of the *vfr* RNA used in electrophoretic mobility shift assay (EMSA) experiments are shown, spanning from the transcriptional start and partially through the *vfr* open reading frame (ORF). The leader sequence of the *vfr* transcript (Fuchs et al., 2010) possesses two putative Hfq-binding sites (blue boxes) and a RsmA-binding site (green box) overlapping the predicted Shine–Dalgarno sequence (red italics) upstream of the translational start site (underlined italics). **(B)** RNA EMSA shows that RsmA does not bind to the *vfr* RNA when RsmA is present alone. Dimeric molar excess relative to RNA; U, unbound RNA. **(C)** The *vfr* RNA produces three distinct band shifts (open arrowheads 1, 2, and 3) with increasing concentrations of Hfq. Hexameric molar excess relative to RNA. **(D)** The presence of RsmA causes all Hfq-shifted bands to super-shift (filled arrowheads 1+, 2+, and 3+), indicating that RsmA binds to Hfq-bound RNA. Note that the RsmA concentration used (18 molar excess of RNA) is incapable of producing a shift when present alone **(B)**. **(E)** A GG→CC substitution within the RsmA-binding site abolishes RsmA super-shifts but does not alter Hfq binding. **(F)** RsmA can bind to the truncated form of the *vfr* RNA that lacks the region immediately upstream of the RsmA-binding site that encompasses the two Hfq-binding sites. Note that this indicates removal of an inhibitory structure that otherwise blocks RsmA binding unless counteracted by binding of Hfq.

Given the evidence that RsmA may post-transcriptionally regulate *vfr*, we next examined the binding properties of RsmA to *vfr* mRNA. *P. aeruginosa* RsmA has a binding specificity for the CANGGAYG consensus sequence of its target mRNA (Schulmeyer et al., 2016), which is similar to the *E. coli* CsrA consensus sequences RUACARGGAUGU generated by SELEX (Dubey et al., 2005) and AUGGAUG generated by CLIP-Seq (Potts et al., 2017). The transcriptional start site for *vfr* lies 153 bases upstream of the translational start site (Fuchs et al., 2010). Inspection of the *vfr* leader sequence identified one putative RsmA-binding site overlapping the likely GGGA Shine–Dalgarno sequence (Figure 4A).

Purified RsmA protein directly binds *psl* mRNA (Irie et al., 2010) in RNA electrophoretic mobility shift assays (EMSAs). However, no RsmA binding was observed in similar assays with *vfr* RNA spanning the leader sequence (Figure 4B). This result led us to consider the possibility that RsmA may require another factor to bind *vfr* mRNA. The RNA-binding chaperone protein Hfq was raised as a candidate for three reasons. First, the small RNA RsmY, which binds multiple RsmA proteins at high affinity to sequester and relieve RsmA-bound mRNAs (Kay et al., 2006), also has the capacity to be bound by Hfq (Sorger-Domenigg et al., 2007), although it was unclear from that study whether co-binding of both proteins occurred. Second, Hfq was identified to bind to *vfr* mRNA by global ChIP-Seq analyses (Kambara et al., 2018). Third, *in silico* analysis of the *vfr* leader sequence revealed two potential Hfq binding sites upstream of the putative RsmA-binding site (Figure 4A).

Biochemical studies, primarily of *E. coli* Hfq, have shown that this hexameric protein complex has at least four regions, which can all be involved in RNA-binding: the proximal face, the distal face, the rim, and the C-terminal tail (Updegrove et al., 2016). *P. aeruginosa* and *Pseudomonas putida* Hfq have shortened C-termini compared with *E. coli* Hfq and may lack C-terminal tail-binding altogether (Milojevic et al., 2013). The proximal site preferentially binds to U-rich RNA sequence (Møller et al., 2002), the distal site binds to A-rich (ARN)_*n*_ triplet repeats (Link et al., 2009), and the rim associates with UA-rich regions (Schu et al., 2015). The two potential Hfq-binding sites found within the *vfr* leader sequence (Figure 4A) represent one A-rich ARN repeat region and one U-rich region.

RNA EMSA analyses show that *P. aeruginosa* Hfq binds *vfr* RNA, resulting in three distinct band shifts (Figure 4C) indicative of two or more binding sites. Because WT *P. putida* Hfq showed identical binding patterns to *P. aeruginosa* Hfq on *vfr* RNA (Supplementary Figure S3A), we took advantage of previously characterized derivatives of *P. putida* Hfq, namely, a distal site mutant (Hfq_Y25D_; A-rich ARN-binding deficient) and a proximal site mutant (Hfq_K56A_; U-rich-binding deficient) (Madhushani et al., 2015) to further analyze Hfq binding to the *vfr* RNA. As seen in Supplementary Figure S3B, Hfq_Y25D_ only recapitulates the second band shift, while Hfq_K56A_ only recapitulates the first band shift. Notably, the third band shift is absent with both mutant derivatives of Hfq. We conclude that the first shift is caused when the ARN repeats are bound by Hfq (Hfq-site 1 in Figure 4A), the second shift is caused by Hfq binding to the U-rich region of the *vfr* leader sequence (Hfq-site 2 in Figure 4A), and the third shift occurs when both sites are occupied simultaneously.

Having ascertained that Hfq does bind *vfr* RNA, we next added RsmA and Hfq simultaneously to the *vfr* RNA. As seen in Figure 4D, this results in super-shifting of all three Hfq band shifts in the presence of RsmA, suggesting that binding of Hfq to either of its target sites is sufficient to allow RsmA binding. Because the GG doublet within the consensus sequences for RsmA and CsrA binding is essential for their abilities to bind cognate target RNAs (Dubey et al., 2005; Irie et al., 2010), we made a CC substitution of the GG doublet within the predicted RsmA-binding region of *vfr* RNA. No RsmA-mediated super-shifting of Hfq band shifts was detected with the CC substituted RNA (Figure 4E).

We infer from the findings above that Hfq binding is a prerequisite for RsmA to bind to its target within *vfr* mRNA. As expanded upon in the *Discussion*, such a prerequisite is indicative of an inhibitory secondary mRNA structure that blocks RsmA binding in the absence of Hfq binding. To test this idea, we generated a truncated version of the *vfr* RNA that lacks the upstream region that encompasses the two Hfq sites and thus does not bind Hfq (Supplementary Figure S4). RsmA can bind to the truncated version of *vfr* RNA independent of Hfq (Figure 4F). Together with the data in preceding sections, these results lead us to conclude that (1) the direct binding of RsmA on *vfr* RNA is prevented by a mRNA structure that can be counteracted by binding of the RNA chaperone protein Hfq, and (2) RsmA is an indirect regulator of biofilm polysaccharide locus *pel* and motility, through its Hfq-assisted action on *vfr* propagated through a regulatory cascade from Vfr to FleQ.

### Loss of Hfq Abolishes *vfr* Expression

**FIGURE 5:**
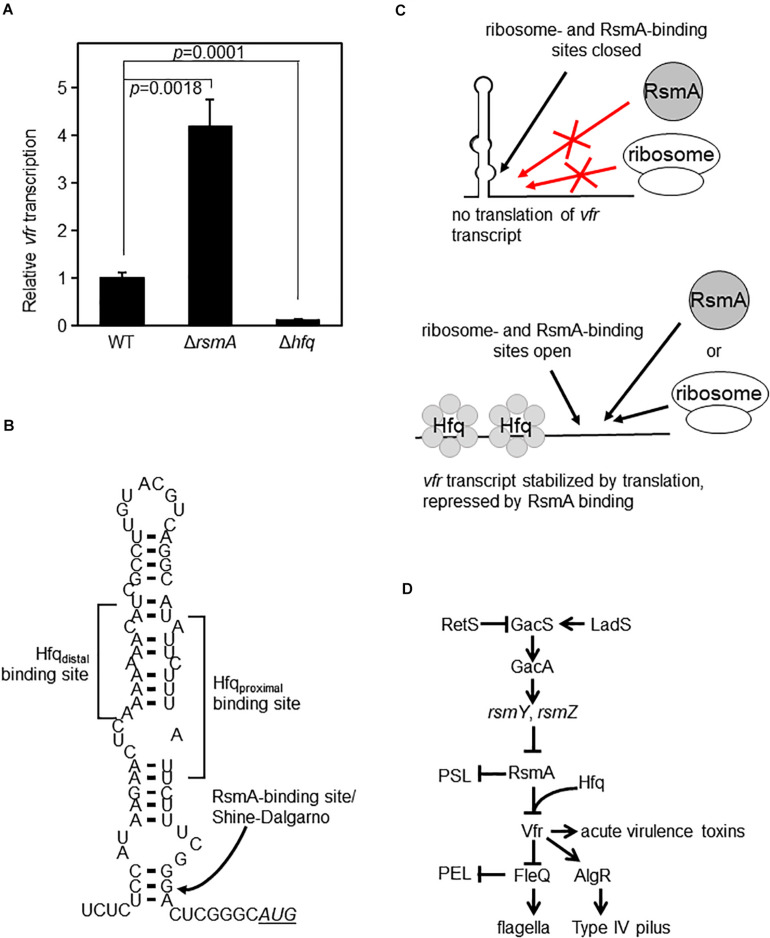
Hfq and RsmA affect *vfr* expression. **(A)** Single-copy transcriptional *lacZ* fusion constructs show that *vfr* transcript levels are increased in the Δ*rsmA* background (as in Figure 1C) but are essentially abolished in the Δ*hfq* mutant background. The *y*-axis values are expressed as relative to WT as 1. **(B)** RNA secondary structure of the leader sequence region of *vfr* as predicted by mFOLD (Zuker, 1989, 2003). The locations of *in silico*-detected Hfq- and RsmA-binding sites are indicated. Note that the RsmA-binding site located at the base of the stem-loop structure overlaps with the Shine–Dalgarno sequence of the ribosome binding site. The AUG start codon is shown in underlined italics. **(C)** Schematic illustration of a model for Hfq- and RsmA-mediated regulation of *vfr*. In the absence of Hfq, the stem-loop structure shown in panel **(B)** blocks binding of both RsmA and ribosomes, resulting in a decrease of *vfr* expression as seen in panel **(A)**. Upon Hfq binding of *vfr* RNA, the stem-loop structure shown in panel **(B)** is disrupted, exposing the ribosome- and RsmA-binding sites. RsmA-binding then directly blocks the Shine–Dalgarno sequence, preventing ribosome access and thereby inhibiting translation. *In vivo*, the untranslated *vfr* mRNAs are presumed to be subsequently rapidly processed for degradation. **(D)** Simplified overview of the Gac/Rsm pathway that controls RsmA levels and pertinent parts of its downstream regulon.

Based on our biochemical findings, we rationalized that since Hfq enables RsmA to bind to *vfr* mRNA, Hfq would serve as a repressor of *vfr*. The prediction from this model is that *vfr* expression would be up-regulated in a Δ*hfq* mutant. We therefore constructed an in-frame deletion mutant of *hfq*. Because Hfq (like RsmA) is a global regulator, its absence can cause pleotropic effects. As previously reported by other laboratories (Sonnleitner et al., 2003; Hill et al., 2019), lack of Hfq in PAO1 causes growth defects (Supplementary Figure S5A). These defects are complemented by WT Hfq (Supplementary Figure S5B). Upon measurement of *vfr* transcriptional activities of equivalent exponential phase cultures, we found that contrary to initial expectations, lack of Hfq led to a complete abolition of *vfr* expression (Figure 5A and Supplementary Figure S6). We attribute this lack of *vfr* expression in the Hfq null strain to be the result of likely indirect effects on translation and RNA stability. We propose that Hfq enables RsmA to bind and inhibit *vfr* at the post-transcriptional level as depicted in Figures 5B,C. Within this model, Hfq is required to disrupt a large inhibitory stem-loop structure formed through complementary sequences that encompass the A-rich Hfq distal binding site and the U-rich Hfq proximal binding site. This large stem loop also blocks ribosome access to the Shine–Dalgarno sequence due to dsRNA formation at the base of the structure. The lack of Hfq in Δ*hfq* strain would thereby result in an inability of the stem-loop structure to release the ribosome binding site for translation, irrespective of the presence or absence of RsmA.

## Discussion

In this study, we report that *Pseudomonas aeruginosa* RsmA requires the RNA chaperone Hfq to assist its binding to *vfr* mRNA to initiate a regulatory cascade that ultimately impacts *pel* biofilm polysaccharide genes. The requirement for Hfq was unexpected, since all other biochemical analyses of RsmA/CsrA family members document unaided binding to their target RNAs (Baker et al., 2002; Dubey et al., 2003; Wang et al., 2005; Lapouge et al., 2007; Irie et al., 2010; Patterson-Fortin et al., 2013; Yakhnin et al., 2013; Ren et al., 2014).

We identified two independent Hfq-binding sites upstream of the RsmA-binding motif, which overlaps the ribosome-binding site of *vfr* (Figure 4A). Based on a mFOLD-predicted secondary structure (Zuker, 1989, 2003), a large hairpin loop base-pairs the identified U-rich and A-rich Hfq-binding sites, resulting in a dsRNA structure that would predictively block both RsmA and the ribosome from accessing the mRNA (Figure 5B). As depicted in Figure 5C, the simplest model that we propose from our findings is that Hfq binding is required to open the stem loop to expose the RsmA-binding site. Subsequent binding of RsmA would directly block the Shine–Dalgarno sequence, preventing ribosome access and thereby inhibiting translation. We also provide evidence that *vfr* mRNA is more stable in the absence of RsmA (Figure 1F), indicating that Hfq- and RsmA-bound *vfr* mRNA may undergo more rapid degradation. mRNAs that are not bound by ribosomes are known to be degraded more rapidly (Deneke et al., 2013). Alternatively and not mutually exclusively, it was previously suggested that Hfq may be able to directly recruit RNase E for the degradation of bound RNA in *Escherichia coli* (Morita et al., 2005), and it is possible that a similar mechanism contributes to changes in *vfr* mRNA stability.

Due to its histidine-rich C-terminus, *E. coli* Hfq is a common contaminant of His-tagged proteins purified by nickel affinity chromatography after over-expression in *E. coli*, and visually undetectable [on sodium dodecyl sulfate–polyacrylamide gel electrophoresis (SDS-PAGE)] Hfq levels can critically influence *in vitro* analyses of RNA binding (Milojevic et al., 2013; Moreno et al., 2015). Because the proteins used in this study were purified after over-expressing them in either *P. aeruginosa* (RsmA) or a Hfq null strain of *E. coli* (Hfq proteins), our analyses were not complicated by this issue. However, a large majority of analyses performed in past publications use C-terminal His-tagged RsmA/CsrA proteins derived from over-expression in *E. coli*, raising the possibility of *E. coli* native Hfq contamination. Given that Hfq binds *vfr* RNA with apparent high affinity (Figure 4C and Supplementary Figure S3), it is plausible that Hfq-assisted RsmA binding may be prevalent. Although such a possibility remains to be experimentally verified, it provokes the observation that comparison of *P. aeruginosa* transcriptomic studies done for RsmA, Hfq, and Vfr regulons identifies numerous overlaps of genes that were differentially expressed between WT and the respective null mutants (Wolfgang et al., 2003; Goodman et al., 2004; Burrowes et al., 2006; Brencic and Lory, 2009; Sonnleitner et al., 2018). Overlaps were also seen between the Hfq and CsrA regulons of *E. coli* (Potts et al., 2017), suggesting that the interplay between the two RNA-binding regulators is not unique to *P. aeruginosa*.

A diverse range of phenotypes have been associated with RsmA functions in *P. aeruginosa* (Vakulskas et al., 2015), but phenotypic observations do not distinguish direct regulatory activities from indirect effects through regulatory cascades. Our identification of Hfq and RsmA action on *vfr* mRNA resulted from an initial aim to determine whether RsmA directly targeted *pel* RNA, as is the case for *psl* RNA (Irie et al., 2010). Our backtracking to trace the ultimate cause of RsmA effects on *pel* expression revealed a regulatory pathway originating from Vfr to the transcriptional regulator FleQ and from there to *pel* (Figure 5D).

This work places the global post-transcriptional regulators RsmA and Hfq as a hub at the center of upstream and downstream regulatory pathways and thus provides a conceptually new framework to evaluate and interpret past work. The two-component GacS/GacA system lies at the top of the hierarchy that controls RsmA-mediated regulation. Although the environmental cue for activation is still unknown, GacS activity is modulated through interactions with RetS and LadS (Goodman et al., 2009; Kong et al., 2013; Chambonnier et al., 2016). GacA, in turn, controls the production of the small RNAs—*rsmY* and *rsmZ*—which possess multiple high-affinity binding sites to titrate RsmA away from target mRNAs (Heurlier et al., 2004; Sonnleitner et al., 2006). In light of a report indicating that Hfq also binds to *rsmY* (Sorger-Domenigg et al., 2007), it appears plausible that these small RNAs may also require Hfq to assist their functions.

In some previous studies, RsmA was proposed to be a positive regulator of *vfr* due to decreased cytotoxicity in Δ*rsmA* mutants (Marden et al., 2013). A subsequent study proposes that Hfq assists sRNAs to inhibit RsmA activity, resulting in increased cytotoxicity in Δ*hfq* mutants since RsmA is de-repressed in this model (Janssen et al., 2020). Our experiments show that RsmA negatively regulates *vfr* post-transcriptionally. To ensure that our phenotypes further correlate with our model, we performed cytotoxicity assays on tissue culture cells, and in our hands, we found Δ*rsmA* mutant to have increased cytotoxicity toward tissue culture cells than WT PAO1 (Figure 3), and *vfr* expression is abolished in Δ*hfq* (Figure 5A). It is unclear why our results are incongruous with some publications, however, it should be noted that in the studies that observe decreased cytotoxicity with a Δ*rsmA* strain, the *P. aeruginosa* background is PA103 whereas this study uses PAO1. Our data are consistent with a publication from another group, which reported that Δ*rsmA* is more cytotoxic in the PAO1 background (Mulcahy et al., 2006). PA103 is frequently used for acute infection studies due to its high toxigenicity (Liu, 1966), and it is possible that its regulatory networks are wired differently from PAO1 due to its high adaptation toward pathogenicity. The precise roles of sRNAs and possible strain-to-strain differential expression patterns in the context of Hfq and RsmA remain to be investigated further.

Numerous processes are associated with RsmA in *P. aeruginosa*, including biofilm formation, motility, and acute virulence (Mulcahy et al., 2006; Intile et al., 2014). Many of these “RsmA-regulated factors and phenotypes” are likely to be indirectly regulated through Vfr- and/or FleQ-initiated regulatory cascades. Based on our findings, it will be crucial to determine and distinguish direct versus indirect regulatory routes to gain a greater understanding of the RsmA regulon.

## Materials and Methods

### Bacterial Strains and Growth Conditions

Supplementary Table S1 lists the bacterial strains used in this study. *Escherichia coli* and *Pseudomonas aeruginosa* strains were grown in lysogeny broth (LB) Lennox composition at 37°C unless specified otherwise. VBMM citrate medium (Hoang and Schweizer, 1997) was used for selecting *P. aeruginosa* post-conjugation. For *E. coli*, the following antibiotics concentrations were used: 50 μg⋅ ml^–1^ of carbenicillin, 10 μg⋅ ml^–1^ of gentamicin, and 10 μg⋅ ml^–1^ of tetracycline. For *P. aeruginosa* strains, 300 μg⋅ ml^–1^ of carbenicillin, 100 μg⋅ ml^–1^ of gentamicin, and 100 μg⋅ ml^–1^ of tetracycline concentrations were used. Sucrose counter-selection for plasmids carrying the *sacB* gene used in *P. aeruginosa* strain constructions was performed by streaking colonies on LB agar (no salt) supplemented with 10% w/v sucrose. Plates were incubated at 30°C for 24 h, after which the counter-selected colonies were confirmed for the loss of antibiotic resistance and mutations confirmed by PCR for double-crossover genomic mutants.

### Strain Constructions

#### Transcriptional Fusion Constructions

β-Galactosidase transcriptional fusion constructs were generated in a single copy on the chromosome of *P. aeruginosa* strains via integration into the chromosomal *attB* site as previously published (Irie et al., 2010). In brief, promoter regions were PCR amplified using oligonucleotides in Supplementary Table S2. PCR products were ligated between the *Eco*RI and *Bam*HI sites of mini-CTX *lacZ* (Supplementary Figure S7). These plasmids were then introduced into *P. aeruginosa* by conjugation. After double site recombination, plasmid backbones were removed by FLP recombinase, and then the strains were cured of the pFLP2 plasmid by sucrose counter-selection (Hoang et al., 2000).

#### In-Frame *hfq* Mutant Construction

Using the same background strain is important in *P. aeruginosa* research, since there is great heterogeneity between PAO1 strains of different laboratories (Klockgether et al., 2010; Chandler et al., 2019). Therefore, we generated Δ*hfq* derivatives in the same PAO1 background as used in the corresponding author’s studies for the past 14 years.

To make an in-frame deletion mutant of *hfq* in different genetic backgrounds, we first attempted to use the suicide plasmid pME3087Δ*hfq* (Sonnleitner et al., 2017). However, even after repeated attempts, we were unable to generate the mutant in a Δ*rsmA* background strains, which are already growth defective (Irie et al., 2010). Therefore, we constructed a Δ*hfq* construct carried on the suicide vector pEX18Gm that has previously successfully been used in Δ*rsmA* strains (Irie et al., 2010) and has the additional advantage of the plasmid containing a gentamicin resistance cassette instead of tetracycline (Hoang et al., 1998). We used pME3087Δ*hfq* as the template to create pEX18Gm:Δ*hfq* to keep our resulting Δ*hfq* mutant constructs consistent with the previous publications. In brief, following the circular polymerase extension cloning (CPEC) protocol (Quan and Tian, 2009, 2011), we first PCR amplified the Δ*hfq* fragment using the primer pair CPEC pEX18Gm H71 and CPEC pEX18Gm I71 (Supplementary Table S2) with pME3087Δ*hfq* as template (70°C annealing temperature, 20-second extension time). The plasmid backbone fragment was amplified using the primer pair CPEC pEX18Gm *Bam*HI and CPEC pEX18Gm *Eco*RI (Supplementary Table S2) with pEX18Gm as template (71.6°C annealing temperature, 60-second extension time). The two fragments were fused and circularized by the second round of PCR that uses a slow ramp anneal of 70 to 55°C at 0.1°C⋅ s^–1^. The product was transformed into DH5α, and the correct configuration of the plasmid was confirmed prior to introduction into S17-1 λ*pir* for conjugation into *P. aeruginosa*. Single-site recombinants were selected on gentamicin VBMM plates. Sucrose plates were used for counter-selection of second-site recombinants. The resulting colonies were screened by PCR using H71 and I71 primer pairs (Supplementary Table S2), where half resulted in WT alleles and half were Δ*hfq.* This screening was facilitated by the fact that Δ*hfq* mutants always formed small colonies due to their growth deficiencies (Supplementary Figure S5).

#### Vfr Over-Expression Construct

Vfr over-expression plasmid pUCP18:*vfr* (Supplementary Figure S8) was constructed by ligating *Eco*RI/*Bam*HI double-digested pUCP18 shuttle vector (Schweizer, 1991) and *Eco*RI/*Bam*HI digested PCR product of primer pairs *vfr* for1 and *vfr* rev1 (Supplementary Table S2) generated using PAO1 genomic DNA as template. The verified plasmid was transformed into *P. aeruginosa* by electroporation (Choi et al., 2006).

#### Hfq Over-Expression Strains

Hfq over-expression strains were made by introducing pME4510*hfq*_*Flag*_ (Sonnleitner et al., 2017) into *P. aeruginosa* by electroporation (Choi et al., 2006).

### Growth Curves

*Pseudomonas aeruginosa* strains were first grown as shaken LB liquid cultures overnight and then diluted to A_600_ ≈ 0.02. Diluted bacteria were deposited into the wells of 96-well plates in triplicates. Growths were monitored in a Synergy^TM^ Mx microplate reader (BioTek) with the chamber temperature at 37°C and maximum plate orbital shaking/agitation speed setting. A_600_ measurements were recorded every 15 min.

### β-Galactosidase Assays

Quantitative β-galactosidase activities were assayed using Galacto-Light Plus kit (Thermo-Fisher) as previously published (Lequette et al., 2006). *P. aeruginosa* cultures were grown at 37°C to exponential phase and lysed using chloroform as previously described (Irie et al., 2010). β-Galactosidase activity units were normalized to total proteins per milliliter as determined using Bradford assay reagents (Bio-Rad). Assays were performed in biological triplicates each in technical triplicates.

### Quantitative Real-Time PCR and RNA Stability Analyses

Real-time PCR was performed as previously described (Irie et al., 2012), using the oligonucleotides listed in Supplementary Table S2. For RNA stability experiments, exponential phase *P. aeruginosa* cultured in VBMM citrate at 37°C were treated with 200 μg⋅ ml^–1^ of rifampicin (Lory, 1986). RNAs were extracted from 1 ml of the cultures at various time points as previously described (Irie et al., 2010) after the addition of rifampicin. RNA extractions were performed using the RNeasy Mini Kit (Qiagen) after treating the harvested cells with RNAprotect (Qiagen). Genomic DNA was removed using DNase I (Promega), and removal confirmed by PCR using primers designed against the *rplU* gene (Supplementary Table S2). SuperScript III First-Strand Synthesis (Invitrogen) with random hexamers was used to synthesize cDNA as per manufacturer’s protocol. Quantitative real-time PCR was performed using SYBR Green PCR Master Mix (Thermo/Applied Biosystems). The internal control gene used was *ampR*. All experiments were done in biological quadruplicates.

### Motility Assays

Motility assays were performed essentially as previously described (Shrout et al., 2006). For swimming motility, LB-Lennox plates containing 0.3% Bacto agar were inoculated with overnight cultures and a sterile inoculation needle, ensuring the needle tip was inserted approximately halfway into the agar but not to the plastic petri dish bottom, and incubated for 24 h at 30°C. Swim ring diameters were measured for quantitation. For twitching motility, LB-Lennox plates containing 0.5% Bacto agar were inoculated by inserting the inoculation needle through the agar until it touched the plastic bottom. Plates were incubated for 72 h at 30°C. Subsequently, the agar was peeled off, and the plates were stained with 1% w/v crystal violet to visualize the twitching zone diameters prior to measurement (Déziel et al., 2001). All motility experiments were performed in at least biological triplicates.

### Protein Expression and Purification

Proteins were purified by previously established protocols. Briefly, over-expressed RsmA-His_6_ was purified from a 4 × 1-L cultures of *P. aeruginosa* PAO1 bearing pUCP18:*rsmA*-His_6_ (Irie et al., 2010), while all Hfq-His_6_ proteins were purified from a 400 ml culture of Hfq null derivative of *E. coli* BL21(DE3) (Madhushani et al., 2015) carrying P_*T7*_ expression plasmids (pVI2344-2346 or pVI2357).

RsmA and Hfq over-expressing strains were grown in 2× YT broth to stationary or exponential phases, respectively. Cell pellets were re-suspended in lysis buffer (buffer A: 0.3 M of NaCl, 20 mM of Tris–HCl pH 8, 5 mM of imidazole) supplemented with 1 tablet⋅ L^–1^ cOmplete^TM^ EDTA-free protease inhibitor tablet (Sigma). Cells were lysed by Stansted Fluid Power SPCH ultra high-pressure cell disrupter/homogenizer. Cell debris were removed by centrifugation (35,000 rpm, 40 min, 4°C), and the supernatants loaded onto 1 ml of HisTRAP HP (GE Healthcare) columns equilibrated in lysis buffer (buffer A). The column was washed with high salt buffer (buffer B: 5 M of NaCl, 20 mM of Tris–HCl pH 8, 5 mM of imidazole) to remove RNA contamination, and the proteins subsequently eluted with the linear gradient of imidazole (25 to 750 mM) in buffer C (buffer C: 0.5 M of NaCl, 20 mM of Tris–HCl pH 8). Fractions containing the desired proteins were pooled. For RsmA-His_6_, an additional ion exchange step using 5 ml of HiTrap SP HP column (GE Healthcare) equilibrated with buffer D (0.1 M of NaCl, 20 mM of Tris–HCl pH 8) was performed, with RsmA subsequently being eluted with 1 M of NaCl.

All proteins were dialyzed across Slide-a-lyzer 10,000 molecular weight cutoff (MWCO) cassettes (Thermo Fisher) against two lots of 2 L dialysis buffer (40 mM of Tris–HCl pH 8, 600 mM of NaCl, 2 mM of EDTA) at 4°C for 2 days prior to concentration using 10-MWCO Centricon (Amicon). Protein concentrations were determined using the bicinchoninic acid (BCA) Protein Assay Kit (Pierce/Fisher), and purity was confirmed by SDS-PAGE. Proteins were stored at −20°C in final storage buffer (20 mM of Tris–HCl pH 8, 300 mM of NaCl, 1 mM of EDTA, 40% glycerol) until use.

### RNA Synthesis

Wild-type and mutant *vfr* mRNAs were generated using Ambion’s MEGAscript kit as recommended for *in vitro* transcription from the P_*T7*_ promoter of linearized plasmids. Reactions (total 40 μl) containing 1 μg of *Eco*RV-linearized pVI2358, pVI2359, or pVI2360 were incubated for 6 h prior to 15-min DNase I treatment. Reactions were terminated by adding 230 μl of RNase-free H_2_O and 30 μl of stop solution (5 M of ammonium acetate; 100 mM of EDTA). The resulting 223-nt RNAs (encompassing coordinates −106 to +116 relative to the A of the initiation codon of *vfr*) for WT and GG→CC versions of *vfr* RNAs and 135-nt RNA for the truncated version of *vfr* RNA (encompassing coordinates −16 to +116 relative to the A of the initiation codon of *vfr*) were extracted twice with phenol:chloroform:IAA (25:24:1) and once with chloroform prior to precipitation and resuspension in 50 μl of RNase-free H_2_O.

### RNA Electrophoretic Mobility Shift Assays

Reactions (total 10 μl) contained 32 nM of *in vitro* transcribed RNA with the indicated concentrations of RsmA and/or Hfq. RsmA and Hfq molarities given here are for the dimeric and hexameric complexes, respectively. RNA was first heated at 80°C for 5 min and then immediately chilled in an ice-water bath. Additional final reaction mixture components were as follows: 10 mM of HEPES pH 7.9, 2 mM of MgCl_2_, 90 ng of yeast total RNA (Fisher), 4U of RNasin, and 35 mM of KCl. Binding reactions were performed at 20°C for 40 min prior to adding 2.5 μl of loading buffer (40% sucrose). Reactions were placed on ice and analyzed on 5% TBE Criterion pre-cast gels (Bio-Rad) with electrophoresis at 100 V for ∼130 min at 4°C. Gels were stained at room temperature with 1:10,000 TBE-diluted SYBR Gold (Fisher) in a light-protected container with agitation. Images were captured using GE Healthcare Typhoon.

### Tissue Culture Infections

Human RPE cells were grown in DMEM/F12 (Corning DMEM Hams F-12 50/50 Mix), supplemented with 10% of fetal bovine serum and 3% of sodium bicarbonate solution (Gibco) at 37°C with 5% CO_2_. Prior to infection, 2.5 × 10^5^ cells⋅ ml^–1^ of RPE were seeded in 24-well plates to reach ∼80% confluence (microscopically verified) in 24 h. Media were gently aspirated from the top of the cells, and each well was washed using antibiotic-free media. Overnight LB liquid culture-grown bacteria were diluted in DMEM/F12 to achieve the multiplicity of infection (MOI) of 50:1. Infected cells were then incubated at 37°C for 24 h. Bacterial numbers were enumerated by plating serial dilutions onto LB agar and counting colonies at 0 (to verify MOI) and 24 h post-infection.

### Lactate Dehydrogenase Release Assay

Supernatants were recovered from each well, and LDH activities were determined using the LDH kit (Sigma) following the manufacturer’s instructions. Signal outputs were read using a 96-well plate reader (BioTek).

### Statistics

Presented data are the mean with all error bars representing standard deviations of the data collected. Two-tailed *p*-values were calculated from Student’s *t*-test using the GraphPad Prism software. Data are statistically significant if *p* < 0.025.

## Data Availability Statement

The datasets generated for this study are available on request to the corresponding author.

## Author Contributions

ALM (infection studies), VS (*in vitro* RNA synthesis), and YI (all others) designed and performed the experiments and analyzed the data. VM assisted in purification of the RsmA and Hfq proteins. YI and VS wrote the manuscript with input from ALM, VH, VM, and TT. All authors contributed to the article and approved the submitted version.

## Conflict of Interest

The authors declare that the research was conducted in the absence of any commercial or financial relationships that could be construed as a potential conflict of interest.
