# Supplementary material for: Hfq-Assisted RsmA Regulation Is Central to Pseudomonas aeruginosa Biofilm Polysaccharide PEL Expression
Source: Front Microbiol. 2020 Nov 17;11:482585. doi: 10.3389/fmicb.2020.482585 (PMC7705225; doi:10.3389/fmicb.2020.482585)
Supplement: Supplementary file 1 [file Data_Sheet_1.pdf]

Hfq-assisted RsmA regulation is central to *Pseudomonas aeruginosa* biofilm polysaccharide PEL expression

Yasuhiko Irie, Agnese La Mensa, Victoriia Murina, Vasili Hauryliuk, Tanel Tenson, and Victoria Shingler

SUPPLEMENTARY MATERIALS

SUPPLEMENTARY TABLES

Table S1

Bacterial strains and plasmids used in this study.

Strains/plasmids	Description	Source/reference
Pseudomonas aeruginosa		
PAO1	wild type	(Holloway et al., 1979)
Δ*rsmA*	Δ*rsmA*$_{-57\rightarrow 73}$::*FRT*	(Irie et al., 2010)
Δ*pel* Δ*psl*	Double mutant	(Borlee et al., 2010)
Δ*rsmA* Δ*pel* Δ*psl*	Triple mutant	(Irie et al., 2010)
Δ*vfr*	In-frame *vfr* deletion	(Almblad et al., 2015)
Δ*hfq*	In-frame *hfq* deletion	this study
Δ*pel* Δ*psl* Δ*hfq*	Triple mutant	this study
Δ*rsmA* Δ*pel* Δ*psl* Δ*hfq*	Quadruple mutant	this study
PAO1 P_{pel} full::*lacZ* trx	P_{pel}::*lacZ* transcriptional fusion construct	this study
Δ*rsmA* P_{pel} full::*lacZ* trx	P_{pel}::*lacZ* transcriptional fusion construct	this study
Δ*rsmA* Δ*pel* Δ*psl* P_{pel} full::*lacZ* trx	P_{pel}::*lacZ* transcriptional fusion construct	this study
PAO1 P_{fleQ} full::*lacZ* trx	P_{fleQ}::*lacZ* transcriptional fusion construct	this study
Δ*rsmA* P_{fleQ} full::*lacZ* trx	P_{fleQ}::*lacZ* transcriptional fusion construct	this study
PAO1 P_{vfr} full::*lacZ* trx	P_{vfr}::*lacZ* transcriptional fusion construct	this study
Δ*rsmA* P_{vfr} full::*lacZ* trx	P_{vfr}::*lacZ* transcriptional fusion construct	this study
Δ*hfq* P_{vfr} full::*lacZ* trx	P_{vfr}::*lacZ* transcriptional fusion construct	this study
Escherichia coli		
BL21(DE3) -Δ*hfq*::*cat* S1	Hfq null T7 polymerase expression host	(Madhushani et al., 2015)
DH5	cloning strain *endA1 hsdR17*($r_k^-m_k^+$) *supE44 thi-1 recA1 gyrA96 relA1* (φ80*dlac*)	(Hanahan, 1985)

DH5α	cloning strain<br>*endA1 hsdR17*($r_K^- m_k^+$) *supE44 thi-1 recA1 gyrA96 relA1* (φ80*dlac*Δ*(lacZ)M15*)	(Hanahan, 1983)
NEB5α	cloning strain<br>*fhuA2* Δ*(argF-lacZ)U169 phoA glnV44* φ*80*Δ(*lacZ*)M15 *gyrA96 recA1 relA1 endA1 thi-1 hsdR17*	New England BioLabs
S17-1 λ*pir*	conjugation donor<br>*recA pro hsdR* RP4-2-Tc::Mu-Km::Tn7 λ*pir*	(de Lorenzo and Timmis, 1994)
DB3.1	conjugation donor<br>*thi thr leu tonA lacY supE recA*::RP4-2-Tc::Mu Km^R	(Miller and Mekalanos, 1988)

Plasmids

pUC57	Cloning vector; Amp/$Carb^R$	GenScript
pVI2358	pUC57-based plasmid for P_{T7} expression of the *vfr* RNA leader sequence. Insert was custom synthesized (GenScript) as an EcoRI/EcoRV fragment, encompassing the T7 promoter sequence and *vfr*, co-ordinates -106 to +116 relative to the A of the initiation codon.<br>$Carb^R$	this study
pVI2359	As above but with a GG at -9 to -10 exchanged to CC within the *vfr* leader sequence.<br>$Carb^R$	this study
pVI2360	As pVI2358 but with *vfr* co-ordinates -16 to +116 relative to the A of the initiation codon, hence removing the two Hfq sites but retaining the RsmA-binding site.<br>$Carb^R$	this study
pFLP2	FLP recombinase expressing plasmid; Amp/$Carb^R$	(Hoang et al., 1998)
pUCP18	*P. aeruginosa - E. coli* shuttle vector; Amp/$Carb^R$	(Schweizer, 1991)
pRsmA ox	RsmA over-expression plasmid (pUCP18 backbone); Amp/$Carb^R$	(Irie et al., 2010)
pUCP18::*vfr*	Vfr over-expression plasmid; Amp/$Carb^R$	this study
pUCP18::*rsmA*-His_6	RsmA-His_6 over-expression plasmid; Amp/$Carb^R$	(Irie et al., 2010)
pME3087Δ*hfq*	In-frame *hfq* deletion construct; Tet^R	(Sonnleitner et al., 2017)
pEX18Gm	suicide vector; $Gent^R$	(Hoang et al., 1998)
pEX18Gm::Δ*hfq*	In-frame *hfq* deletion construct; $Gent^R$	this study

mini-CTX *lacZ*	*lacZ* transcriptional fusion *attB* integration construction plasmid; Tet^R	(Becher and Schweizer, 2000)
mini-CTX lacZ::P_{pel} full	transcriptional full length *pel* promoter *lacZ* fusion construct; Tet^R	this study
mini-CTX lacZ::P_{fleQ} full	transcriptional full length *fleQ* promoter *lacZ* fusion construct; Tet^R	this study
mini-CTX lacZ::P_{vfr} full	transcriptional full length *vfr* promoter *lacZ* fusion construct; Tet^R	this study
pET3H	T7 promoter expression vector; $Amp/Carb^R$	(Shingler and Pavel, 1995)
pVI2344	P_{T7}-PP *hfq*-His pET3H-based expression plasmid; $Amp/Carb^R$	(Madhushani et al., 2015)
pVI2345	P_{T7}-PP *hfq-Y25D*-His pET3H-based expression plasmid; $Amp/Carb^R$	(Madhushani et al., 2015)
pVI2346	P_{T7}-PP *hfq-K56A*-His pET3H-based expression plasmid; $Amp/Carb^R$	(Madhushani et al., 2015)
pVI2357	P_{T7}-PA *hfq*-His pET3H-based expression plasmid; $Amp/Carb^R$	this study
pME4510	Broad host-range promoter-probe plasmid; $Gent^R$	(Rist and Kertesz, 1998)
pME4510*hfq*$_{Flag}$	Hfq-FLAG over-expression plasmid; $Gent^R$	(Sonnleitner and Bläsi, 2014)

Amp = Ampicillin; Carb = Carbenicillin; Gent = Gentamicin; Km = Kanamycin; Tet = Tetracycline

Table S2

Oligonucleotide primers used in this study. Engineered restriction sites are underlined. Genome co-ordinates are based on the annotation of PAO1 on www.pseudomonas.com (Winsor et al., 2011; Winsor et al., 2016).

Oligonucleotide primer	Used for	Genome co-ordinates	Sequence (5' to 3')
P_{pel} full for	P_{pel} full::*lacZ* construction	3434399-3434376	CGGCGAATTCCTGGTGCGGTTCCTCGCACGCAAC
P_{pel} full rev	P_{pel} full::*lacZ* construction	3433863-3433883	GATCGGATCCACGGCGATTCCTTTCTTGCTG
P_{fleQ} full for1	P_{fleQ} full::*lacZ* construction	1187287-1187306	CGGCGAATTCCTACCAGATGTTCGGATAAG
P_{fleQ} full rev1	P_{fleQ} full::*lacZ* construction	1187609-1187589	GATCGGATCCAAGAGTTTGGTTTCGCGCCAC
P_{vfr} full for1	P_{vfr} full::*lacZ* construction	706950-706934	CGGCGAATTCCTTTCATCGTTCAGACT
P_{vfr} full rev1	P_{vfr} full::*lacZ* construction	706650-706672	GATCGGATCCGGTGTGTGGGTAATAGCTACCAT
vfr for1	pUCP18::*vfr* construction	706905-706887	CGGCGAATTCCAGGGCCCAAGGACAGTAC
vfr rev1	pUCP18::*vfr* construction	706028-706045	GATCGGATCCTCAGCGGGTGCCGAAGAC
H71	PCR confirmation for *hfq* deletion	5549362-5549346	TTTTTTTGGATCCGATACCGAGGTGCGCGC
I71	PCR confirmation for *hfq* deletion	5547601-5547614	TTTTTTTGAATTCGCGGCAGGTGGCGG
CPEC pEX18Gm BamHI	pEX18Gm::Δ*hfq* construction	pEX18Gm template	GGATCCTCTAGAGTCGACCTGCAGG
CPEC pEX18Gm H71	pEX18Gm::Δ*hfq* construction	5549362-5549354	GGTCGACTCTAGAGGATCCGATCCCGAG
CPEC pEX18Gm EcoRI	pEX18Gm::Δ*hfq* construction	pEX18Gm template	GAATTCGTAATCATGGTCATAGCTGTTTCCTGTGTG
CPEC pEX18Gm I71	pEX18Gm::Δ*hfq* construction	5547160-5547167	TGACCATGATTACGAATTCGCGGCAGG
rplU forward	Checking for gDNA contamination in RNA prep	5116619-5116600	CGCAGTGATTGTTACCGGTG
rplU reverse	Checking for gDNA contamination in RNA prep	5116315-5116334	AGGCCTGAATGCCGGTGATC

ampR-F	RT-PCR	4593527-4593511	GCGCCATCCCTTCATCG
ampR-R	RT-PCR	4593473-4593491	GATGTCGACGCGGTTGTTG
pelA-F	RT-PCR	3432090-3432070	CCTTCAGCCATCCGTTCTTCT
pelA-R	RT-PCR	3431973-3431992	TCGCGTACGAAGTCGACCTT
fleQ RT for1	RT-PCR	1187644-1187666	CTGGCAGTCATTCTCAACTTCCT
fleQ RT rev1	RT-PCR	1187706-1187689	TCGCCAATCCTCGCTGTT
vfr RT for1	RT-PCR	706504-706489	GACGGCCGCGAAATGA
vfr RT rev1	RT-PCR	706446-706462	CCCAGCTCGCCGAAGAA

SUPPLEMENTARY FIGURE LEGENDS

Fig. S1 RsmA over-expression complements Δ*rsmA* effects on *vfr* transcript levels.
Single-copy transcriptional *lacZ* fusion constructs show that over-expression of RsmA in a Δ*rsmA* strain background reduces the elevated levels of *vfr* observed in the absence of RsmA. VC = vector control.

Fig. S2 PEL and PSL expressions do not significantly affect swimming motility.
Decreased flagellar-mediated swimming motility of the Δ*rsmA* strains (as shown in Fig. 2A) is not due to the expression of the PEL and PSL polysaccharides; note that Δ*rsmA* Δ*pel* Δ*psl* is less motile than WT and Δ*pel* Δ*psl* strains.

Fig. S3 EMSA analysis of Hfq binding to *vfr* RNA.
A. *P. aeruginosa* Hfq and *P. putida* Hfq bind identically to *P. aeruginosa vfr* RNA. Band shifts are indicated by the open arrow heads 1, 2, and 3 as under Fig. 4. Molar ratios of hexameric Hfq over RNA are indicated; U = unbound RNA.
B. Mutant version of *P. putida* Hfq in the distal binding site (Hfq_{Y25D}) lack the first band-shift, but the second shift is present. Conversely, the proximal site mutant version of *P. putida* Hfq (Hfq_{K56A}) produces the first band but not the second. Note that neither mutant Hfq proteins produce the third band, which is interpreted to result from two Hfq hexamers simultaneously binding to the A-rich site (Hfq-site 1) and U-rich site (Hfq-site 2) per *vfr* RNA (Fig. 4A).

Fig. S4 EMSA analysis of Hfq binding to truncated *vfr* RNA lacking Hfq-binding sites.
Hfq is unable to bind to the truncated version of *vfr* RNA that lacks both Hfq-binding sites 1 and 2. Molar ratios of hexameric Hfq over RNA are indicated; U = unbound RNA

Fig. S5 Effects of *hfq* deletion mutation or over-expression on growth.
A. Growth curves of PAO1 (WT), Δ*hfq*, Δ*pel* Δ*psl*, Δ*rsmA* Δ*pel* Δ*psl*, Δ*pel* Δ*psl* Δ*hfq*, Δ*rsmA* Δ*pel* Δ*psl* Δ*hfq* monitored by 600 nm absorbance (A_{600}) over 12 hours. Mutants in *hfq* exhibit growth defects as highlighted by a slower doubling time and lower maxima as compared to WT. Mutants defective in both *rsmA* and *hfq* exhibit additive defects in growth. Left panel: y-axis plotted in linear scale. Right panel: y-axis plotted in $\log_2$ scale.

B. Growth curves of Δ*hfq* transformed with empty vector pME4510 (VC) or Hfq over-expression plasmid were monitored by A_{600} over 24 hours. Left panel: y-axis plotted in linear scale. Right panel: y-axis plotted in $\log_2$ scale.

Fig. S6 Hfq over-expression complements Δ*hfq* effects on *vfr* transcript levels.
Single-copy transcriptional *lacZ* fusion constructs show that over-expression of Hfq in the Δ*hfq* strain background restores the levels of *vfr* transcripts.

Fig. S7 P_{pel}-, P_{fleQ} -and P_{vfr}-*lacZ* transcriptional fusion constructs.
A. Simplified plasmid map of mini-CTX *lacZ* as published previously (Becher and Schweizer, 2000). The expanded multiple cloning site (MCS) highlights the two restriction sites in blue (EcoRI and BamHI) into which transcriptional fusion fragments (panels B-D) were cloned. *tet* = tetracycline resistance gene; *lacZ* = β-galactosidase gene
B-D. Intergenic regions that were cloned to produce the transcriptional fusion constructs are shown for P_{pel}, P_{fleQ}, and P_{vfr} respectively. The regions between the vertical dashed lines were amplified using the primers (solid black arrows; primer names from Table S2 shown beneath the arrows). The hooked arrows represent the transcriptional start sites and the transcriptional directions of corresponding downstream genes as determined in previous studies (Dasgupta et al., 2002; Fuchs et al., 2010; Baraquet et al., 2012). The specific chromosomal co-ordinate positions are indicated with the numbers corresponding to the *P. aeruginosa* PAO1 genome sequence annotated in www.pseudomonas.com (Winsor et al., 2011; Winsor et al., 2016).
Items in Fig. S7 are not drawn to scale.

Fig. S8 Vfr over-expression (pUCP18::*vfr*) construct
A. Simplified plasmid map of pUCP18 as published previously (Schweizer, 1991). The expanded MCS highlights the two restriction sites in blue (EcoRI and BamHI) into which the *vfr* gene was cloned. *bla* = β-lactamase gene
B. The *vfr* protomer region and ORF between the vertical dashed lines were PCR amplified using the primers (solid black arrows; primer names from Table S2 shown beneath the arrows). The specific chromosomal co-ordinate positions are indicated with the numbers corresponding to the *P. aeruginosa* PAO1 genome sequence annotated in www.pseudomonas.com (Winsor et al., 2011; Winsor et al., 2016).
Items in Fig. S8 are not drawn to scale.

Table S1

Bacterial strains and plasmids used in this study.

Table S2

Oligonucleotide primers used in this study. Engineered restriction sites are underlined. Genome co-ordinates are based on the annotation of PAO1 on www.pseudomonas.com (Winsor et al., 2011; Winsor et al., 2016).

SUPPLEMENTARY REFERENCES

Almblad, H., Harrison, J.J., Rybtke, M., Groizeleau, J., Givskov, M., Parsek, M.R., and Tolker-Nielsen, T. (2015). The Cyclic AMP-Vfr Signaling Pathway in *Pseudomonas aeruginosa* Is Inhibited by Cyclic Di-GMP. *J. Bacteriol.* 197**,** 2190-2200.

Baraquet, C., Murakami, K., Parsek, M.R., and Harwood, C.S. (2012). The FleQ protein from *Pseudomonas aeruginosa* functions as both a repressor and an activator to control gene expression from the *pel* operon promoter in response to c-di-GMP. *Nucleic Acids Res.* 40**,** 7207-7218.

Becher, A., and Schweizer, H.P. (2000). Integration-proficient *Pseudomonas aeruginosa* vectors for isolation of single-copy chromosomal *lacZ* and *lux* gene fusions. *Biotechniques* 29**,** 948-950, 952.

Borlee, B.R., Goldman, A.D., Murakami, K., Samudrala, R., Wozniak, D.J., and Parsek, M.R. (2010). *Pseudomonas aeruginosa* uses a cyclic-di-GMP-regulated adhesin to reinforce the biofilm extracellular matrix. *Mol. Microbiol.* 75**,** 827-842.

Dasgupta, N., Ferrell, E.P., Kanack, K.J., West, S.E., and Ramphal, R. (2002). *fleQ*, the gene encoding the major flagellar regulator of *Pseudomonas aeruginosa*, is σ^{70} dependent and is downregulated by Vfr, a homolog of *Escherichia coli* cyclic AMP receptor protein. *J. Bacteriol.* 184**,** 5240-5250.

De Lorenzo, V., and Timmis, K.N. (1994). Analysis and construction of stable phenotypes in gram-negative bacteria with Tn*5*- and Tn*10*-derived minitransposons. *Methods Enzymol.* 235**,** 386-405.

Fuchs, E.L., Brutinel, E.D., Jones, A.K., Fulcher, N.B., Urbanowski, M.L., Yahr, T.L., and Wolfgang, M.C. (2010). The *Pseudomonas aeruginosa* Vfr regulator controls global virulence factor expression through cyclic AMP-dependent and -independent mechanisms. *J. Bacteriol.* 192**,** 3553-3564.

Hanahan, D. (1983). Studies on transformation of *Escherichia coli* with plasmids. *J. Mol. Biol.* 166**,** 557-580.

Hanahan, D. (1985). "Techniques for transformation of *E. coli*," in *DNA Cloning: A Practical Approach*, ed. D.M. Glover. 1 ed (Oxford: IRL Press Ltd.), 109-136.

Hoang, T.T., Karkhoff-Schweizer, R.R., Kutchma, A.J., and Schweizer, H.P. (1998). A broad-host-range Flp-*FRT* recombination system for site-specific excision of chromosomally-located DNA sequences: application for isolation of unmarked *Pseudomonas aeruginosa* mutants. *Gene* 212**,** 77-86.

Holloway, B.W., Krishnapillai, V., and Morgan, A.F. (1979). Chromosomal genetics of *Pseudomonas*. *Microbiol. Rev.* 43**,** 73-102.

Irie, Y., Starkey, M., Edwards, A.N., Wozniak, D.J., Romeo, T., and Parsek, M.R. (2010). *Pseudomonas aeruginosa* biofilm matrix polysaccharide Psl is regulated transcriptionally by RpoS and post-transcriptionally by RsmA. *Mol. Microbiol.* 78**,** 158-172.

Madhushani, A., Del Peso-Santos, T., Moreno, R., Rojo, F., and Shingler, V. (2015). Transcriptional and translational control through the 5'-leader region of the *dmpR* master regulatory gene of phenol metabolism. *Environ. Microbiol.* 17**,** 119-133.

Miller, V.L., and Mekalanos, J.J. (1988). A novel suicide vector and its use in construction of insertion mutations: osmoregulation of outer membrane proteins and virulence determinants in *Vibrio cholerae* requires *toxR*. *J. Bacteriol.* 170**,** 2575-2583.

Rist, M., and Kertesz, M.A. (1998). Construction of improved plasmid vectors for promoter characterization in *Pseudomonas aeruginosa* and other gram-negative bacteria. *FEMS Microbiol. Lett.* 169**,** 179-183.

Schweizer, H.P. (1991). *Escherichia-Pseudomonas* shuttle vectors derived from pUC18/19. *Gene* 97**,** 109-121.

Shingler, V., and Pavel, H. (1995). Direct regulation of the ATPase activity of the transcriptional activator DmpR by aromatic compounds. *Mol Microbiol* 17**,** 505-513.

Sonnleitner, E., and Bläsi, U. (2014). Regulation of Hfq by the RNA CrcZ in *Pseudomonas aeruginosa* carbon catabolite repression. *PLoS Genet* 10**,** e1004440.

Sonnleitner, E., Prindl, K., and Bläsi, U. (2017). The *Pseudomonas aeruginosa* CrcZ RNA interferes with Hfq-mediated riboregulation. *PLoS One* 12**,** e0180887.

Winsor, G.L., Griffiths, E.J., Lo, R., Dhillon, B.K., Shay, J.A., and Brinkman, F.S. (2016). Enhanced annotations and features for comparing thousands of *Pseudomonas* genomes in the *Pseudomonas* genome database. *Nucleic Acids Res.* 44**,** D646-D653.

Winsor, G.L., Lam, D.K., Fleming, L., Lo, R., Whiteside, M.D., Yu, N.Y., Hancock, R.E., and Brinkman, F.S. (2011). *Pseudomonas* Genome Database: improved comparative analysis and population genomics capability for *Pseudomonas* genomes. *Nucleic Acids Res.* 39**,** D596-600.
