# Supplementary figures and images for: Hfq-Assisted RsmA Regulation Is Central to Pseudomonas aeruginosa Biofilm Polysaccharide PEL Expression
Source: Front Microbiol. 2020 Nov 17;11:482585. doi: 10.3389/fmicb.2020.482585 (PMC7705225; doi:10.3389/fmicb.2020.482585)

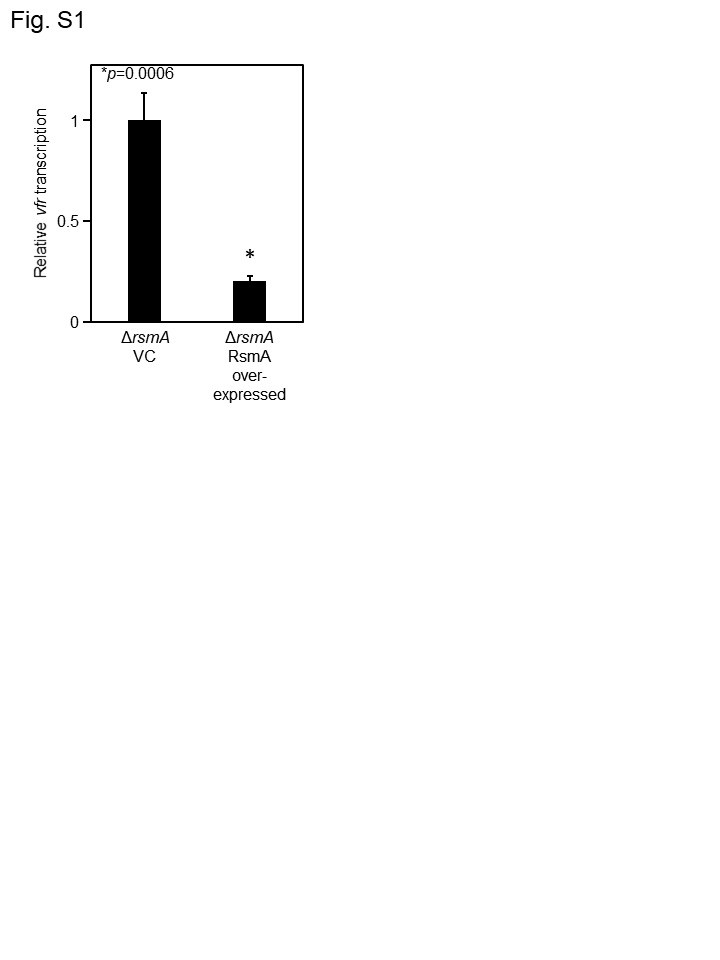

Supplement: Supplementary file 2 [file Image_1.TIF]

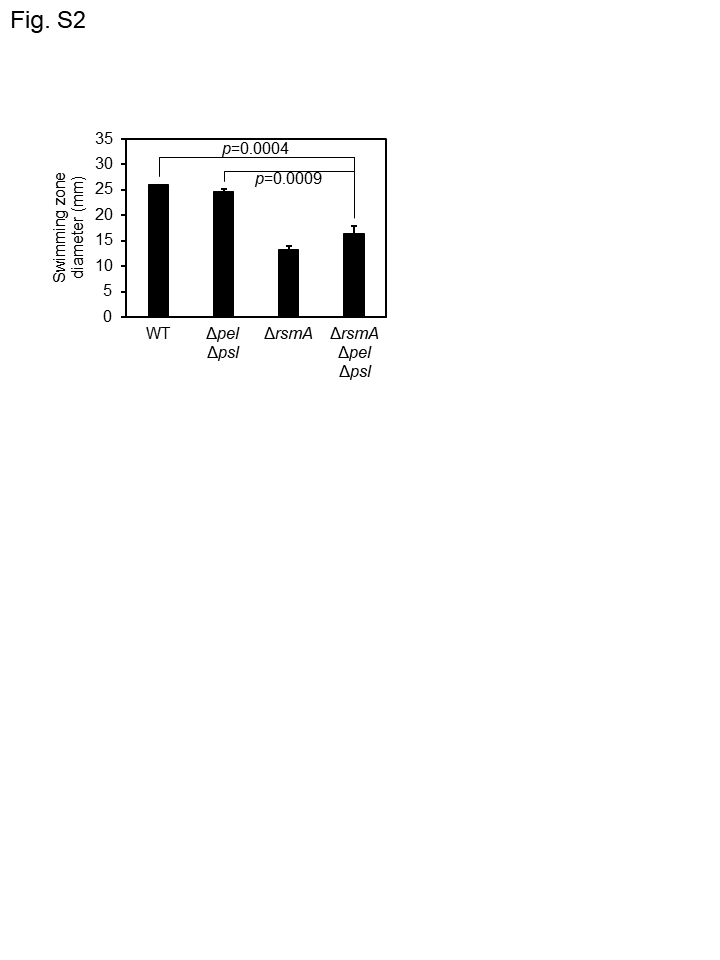

Supplement: Supplementary file 3 [file Image_2.TIF]

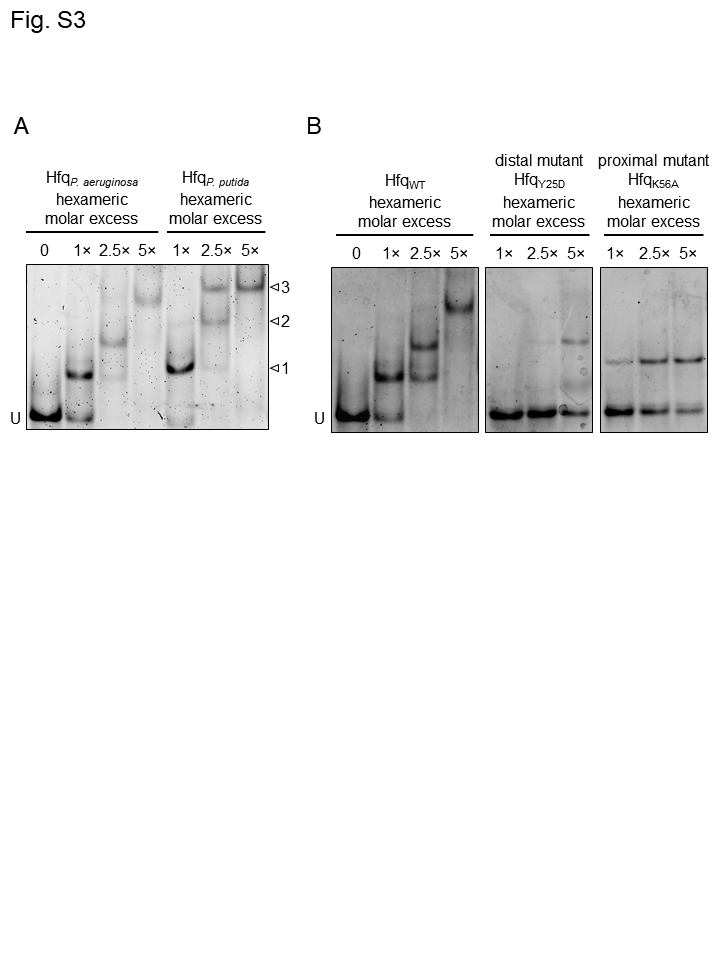

Supplement: Supplementary file 4 [file Image_3.TIF]

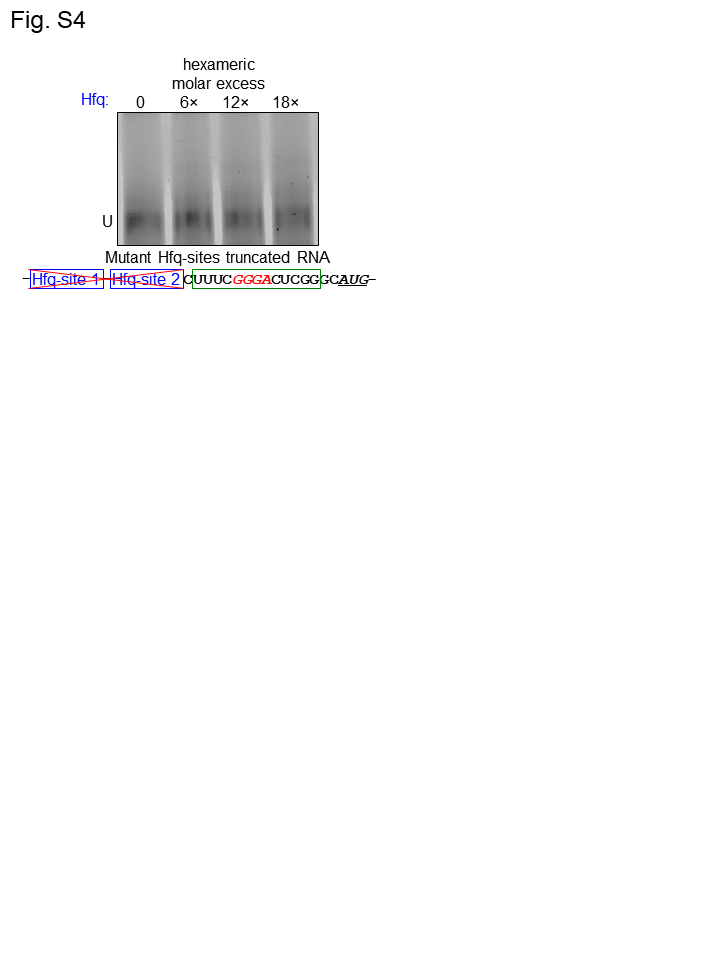

Supplement: Supplementary file 5 [file Image_4.TIF]

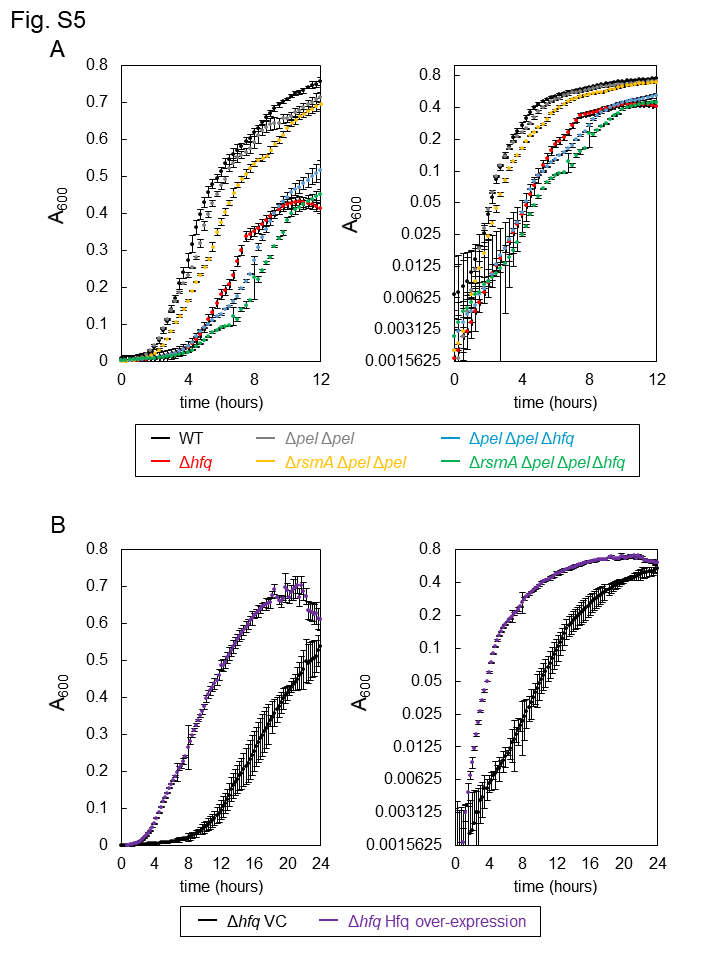

Supplement: Supplementary file 6 [file Image_5.TIF]

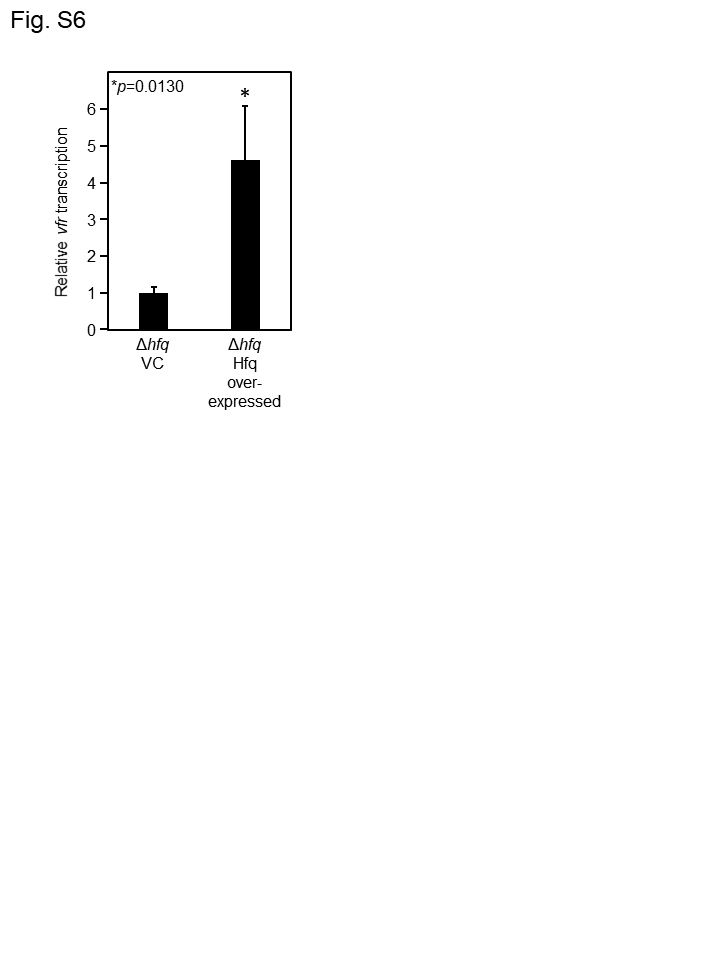

Supplement: Supplementary file 7 [file Image_6.TIF]

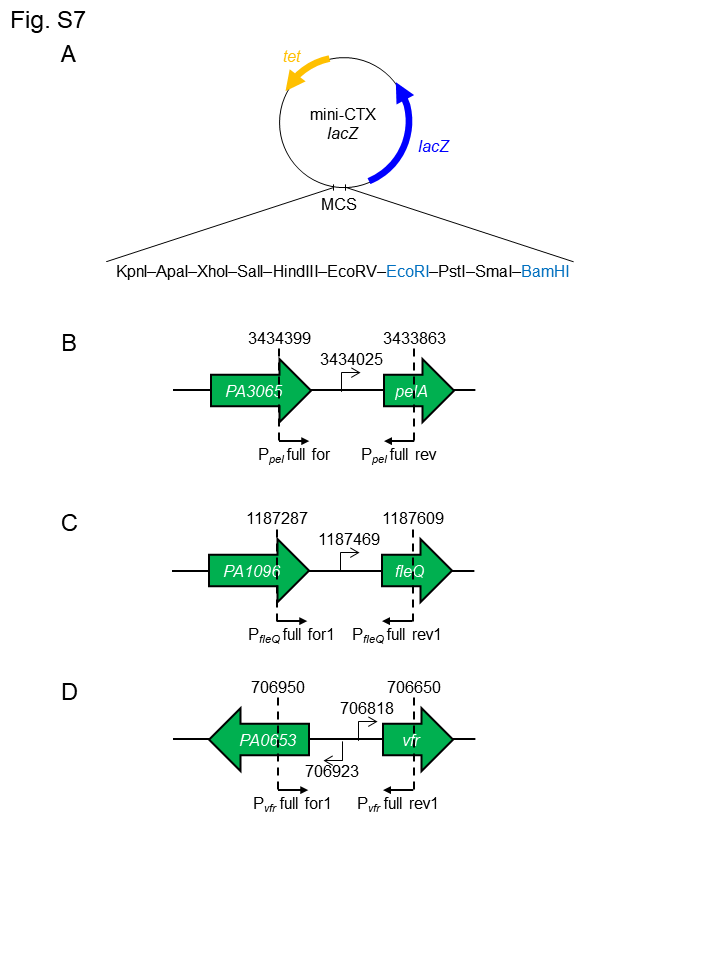

Supplement: Supplementary file 8 [file Image_7.TIF]

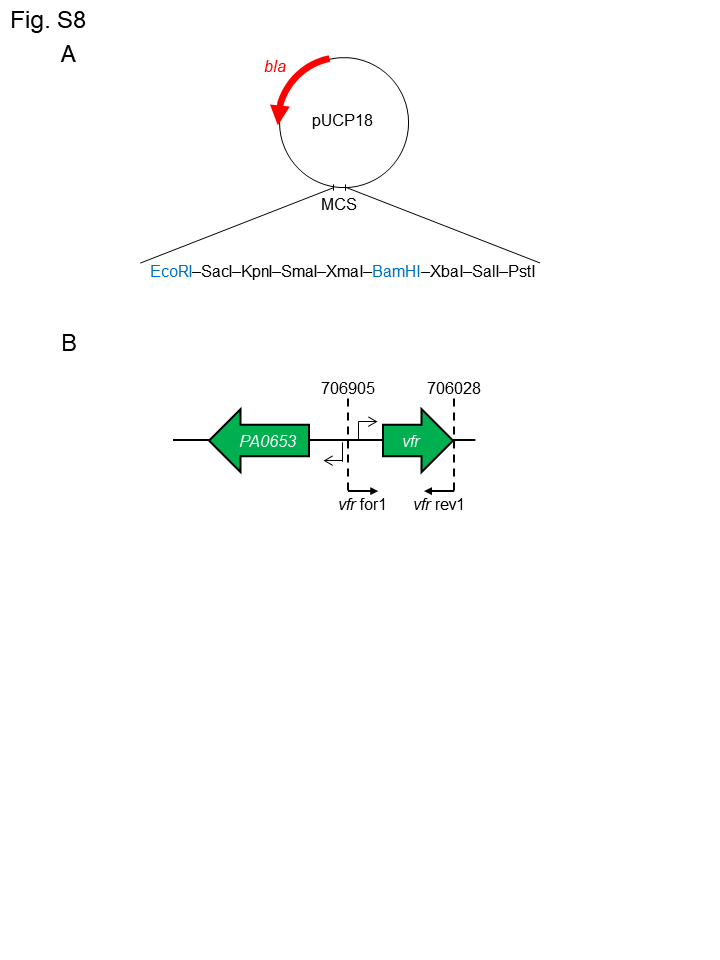

Supplement: Supplementary file 9 [file Image_8.TIF]
